# NEIGHBORHOOD ENVIRONMENTS AND OBESITY: EXPLORING PATHWAYS TO RISK OF CARDIOVASCULAR DISEASE

**DOI:** 10.1093/geroni/igz038.246

**Published:** 2019-11-08

**Authors:** Christine Mair, Amanda Lehning, Nicole Mattocks, Kyeongmo Kim, Roberto Millar, Michele K Evans, Alan B Zonderman, Shari Waldstein

**Affiliations:** 1 University of Maryland, Baltimore County, Baltimore, Maryland, United States; 2 University of Maryland, Baltimore, Baltimore, Maryland, United States; 3 Virginia Commonwealth University, Richmond, Virginia, United States; 4 National Institute on Aging, Baltimore, Maryland, United States

## Abstract

Modifying neighborhood environments to target well-established risk factors for cardiovascular disease may reduce health disparities by complementing clinical services. Prior research, however, includes limited measures of neighborhoods and does not adequately account for individual-level processes known to mediate health outcomes. We combine baseline data from the Healthy Aging in Neighborhoods of Diversity Across the Life Span (HANDLS) dataset with neighborhood-level data to yield a diverse sample of Black and white middle-aged and older residents of Baltimore City (N=2707). We use structural equation modeling to examine associations between neighborhood environments and obesity (BMI>=30), focusing on individual-level mediators. Initial direct associations between neighborhoods and obesity (e.g., presence of businesses, β=-0.062) are mediated by healthcare access and health behaviors. Additional indirect pathways exist through health behaviors (e.g., neighborhood disorder, access to parks). These findings highlight the importance of considering indirect pathways to cardiovascular health promotion among aging adults in different neighborhood contexts.

